# The Impact of Tobacco Smoke Exposure on Wheezing and Overweight in 4–6-Year-Old Children

**DOI:** 10.1155/2014/240757

**Published:** 2014-07-06

**Authors:** Regina Grazuleviciene, Sandra Andrusaityte, Inga Uzdanaviciute, Jolanta Kudzyte, Rimantas Kevalas, Mark J. Nieuwenhuijsen

**Affiliations:** ^1^Department of Environmental Science, Vytautas Magnus University, K. Donelaicio Street 58, 44248 Kaunas, Lithuania; ^2^Department of Children Diseases, Kaunas University of Medicine, Eiveniu Street 2, 50161 Kaunas, Lithuania; ^3^Centre for Research in Environmental Epidemiology (CREAL), Doctor Aiguader 88, 08003 Barcelona, Spain

## Abstract

*Aim*. To investigate the association between maternal smoking during pregnancy, second-hand tobacco smoke (STS) exposure, education level, and preschool children's wheezing and overweight. *Methods*. This cohort study used data of the KANC cohort—1,489 4–6-year-old children from Kaunas city, Lithuania. Multivariate logistic regression was employed to study the influence of prenatal and postnatal STS exposure on the prevalence of wheezing and overweight, controlling for potential confounders. *Results*. Children exposed to maternal smoking during pregnancy had a slightly increased prevalence of wheezing and overweight. Postnatal exposure to STS was associated with a statistically significantly increased risk of wheezing and overweight in children born to mothers with lower education levels (OR 2.12; 95% CI 1.04–4.35 and 3.57; 95% CI 1.76–7.21, accordingly). *Conclusions*. The present study findings suggest that both maternal smoking during pregnancy and STS increase the risk of childhood wheezing and overweight, whereas lower maternal education might have a synergetic effect. Targeted interventions must to take this into account and address household smoking.

## 1. Introduction

Exposure of nonsmoking women to second-hand tobacco smoke (STS) during pregnancy is a serious threat to public health and is a significant cause of damage to the immune system of the fetuses. There is growing evidence that in utero exposure to tobacco smoke adversely affects postnatal lung function and increases the risk of allergic disease [1–6] and also affects infants' physical development and overweight in later life [7–10]. The recent systemic review of wheezing in childhood [11] has suggested that exposure to STS increases the risk of wheezing. Epidemiological studies have revealed that exposure to passive smoke—particularly prenatal or postnatal maternal smoking—is associated with increased risks (from 28% to 70%) of wheeze in children aged 5 to 18 years [12–16]. Some authors reported that maternal smoking increases the risk of allergic sensitization and wheezing only in children with allergic predisposition [16–18] and that interaction of genetic and environmental risk factors has an impact on the incidence and prognosis of wheezing illness during childhood [19–22]. There is evidence that both socioenvironmental factors and tobacco smoke may influence the development of asthma or wheezing [17, 18, 20, 22–24].

Now it has been recognized that exposure to passive smoking is an important risk factor for the incidence of wheeze and asthma during childhood. Less frequently reported associations include children's physical development, overweight, and obesity [25, 26]. The studies of childhood overweight have reported far less consistent results [10, 27–29], and causal relations between exposure to smoking in utero and childhood overweight lack evidence.

There is speculation that the effect of intrauterine tobacco exposure on childhood obesity may depend largely on cigarette smoking during the first trimester, whereas the additional impact of smoking throughout pregnancy might be due to confounding by sociodemographics [8] or residual confounding by genetic and family environmental factors [7, 10]. However, a systematic review of studies reporting on the association between maternal prenatal cigarette smoking and elevated risk for childhood overweight suggested that sociodemographic and behavioral differences between smokers and nonsmokers did not explain the observed association [26]. The review suggested that prenatal smoke exposure led to about 50% increase in the risk of being overweight in childhood. Based on the meta-analysis, the authors' findings showed that children whose mothers smoked during pregnancy were at an elevated risk for overweight (pooled adjusted odds ratio 1.50, 95% CI 1.36–1.65), compared with children whose mothers did not smoke during pregnancy [25, 30, 31]. The findings of a population-based prospective cohort study suggested that direct intrauterine exposure to smoke until late pregnancy leads to different height and weight growth adaptations and an increased risk of overweight and obesity in preschool children [32]. It has been suggested that maternal smoking during pregnancy increases the risk of obesity in the offspring [9, 33]; however, additional studies are needed to assess directly whether smoking in early pregnancy increases long-term risk of obesity-related disorders in children [34]. Only a few studies have explored the impact of sociodemographics factors of STS on preschool children health [5, 35, 36].

In the present study, using individual cohort study data, we investigated the association between STS during pregnancy in nonsmoking and smoking mothers, the individual-level socioeconomic status (SES), and wheezing and overweight in 4–6-year-old children. We hypothesized that the effects would be more apparent in children born to mothers with lower rather than higher level of education.

## 2. Methods

### 2.1. Study Population

The data used in these analyses were collected as part of the PHENOTYPE project (Positive Health Effects of the Natural Outdoor Environment in Typical Populations in Different Regions in Europe) funded by the European Commission Seventh Framework Program [37]. We used survey data on women who were recruited to the pregnant women cohort study during 2007–2009. The first interview was completed during the first trimester of pregnancy. Women reported their age at inclusion (less than 30 years or 30 years and older), education level (low—10 or fewer years, medium and high—more than 10 years), social status (worker, student, unemployed—low; housekeeper, officer—medium; manager, company owner—high), marital status (married or not married), smoking (nonsmoker or smokes at least one cigarette per day), maternal body mass index (BMI) (<25, 25–30, or >30 kg/m2), and other variables. Active maternal smoking at enrollment was assessed in the first questionnaire by asking whether the mother smoked during her pregnancy. The second interview was completed just after childbirth (76%) or by telephone within the first month after delivery (24%), and data on residential history, job during pregnancy, health behavior, and other variables were collected. Individual-level SES predictors were education level and occupation type. Postal questionnaires sent in 2013 at children's ages 4–6 years provided information about children's health. Information collected from the mothers included details on the exposure to tobacco smoke. However, because of the relatively small number of smoking mothers in the sample, we chose not to divide this group into smaller categories.

In this study, we investigated the influence of maternal exposure to domestic cigarette smoke during pregnancy on the prevalence of wheezing and being overweight in the children. We separately analyzed mothers who themselves smoked during their pregnancy from those who did not but were exposed to domestic cigarette smoking. The participants were 1,489 children residing in Kaunas city, Lithuania, who in 2013 were 4–6 years of age and whose parents or guardians responded to questionnaire and agreed to participate in the study. The study was approved by the Lithuanian Bioethics Committee, and parental informed consent was obtained from all participants. Questionnaire responses by parents or guardians were used to categorize children's basic information, medical history, family history, personal habits, children's height and weight, and housing and environmental conditions. Responses to the standardized International Study of Asthma and Allergies in Childhood (ISAAC) questionnaire completed by parents were used to identify children with symptoms of wheezing. Wheezing during the last 12 months was identified by an affirmative response to the question: “Has your child experienced wheezing (whistling sounds in the chest) over the last 12 months?” The outcome measure was wheezing during last 12 months and a variety of social and environmental factors were taken into account.

To assess children's overweight, their BMI was calculated as the ratio of weight (in kg)/height (in m2). To define overweight and obese in our study, we used age group and sex-fixed BMI cutoff points recommended for the evaluation of overweight and obesity in children according to the Childhood Obesity Working Group of the International Obesity Taskforce (IOTF) guidelines [38]. BMI classes were evaluated according to the IOTF: underweight (BMI ≤ 14 kg/m2); normal weight (BMI > 14 kg/m2 and <18 kg/m2); overweight (BMI ≥ 18 kg/m2 and <20 kg/m2); and obesity (BMI ≥ 20 kg/m2). Because of the small number of obese children (*N* = 36), overweight and obese groups were merged in the analysis.

### 2.2. Statistical Analysis

We used chi-square and univariate logistic regression analyses to compare values and frequencies of the baseline characteristics by smoking status of the study subjects. Subsequently, we evaluated the associations among the covariates that are known to be related to an increased risk of wheezing and overweight. Predictor variables whose univariate test showed a statistically significant association (*P* < 0.05) with the outcome—or those that changed the adjusted odds ratios (aOR) by 10% or more—were retained for inclusion in multivariate logistic regression analyses.

Multivariate logistic regressions were used to assess the relationship between the prevalence of wheezing in STS-exposed and nonexposed women's children, adjusting for the mothers' education level, the children's sex, birth weight, and antibiotic use during the first year of life; and for overweight adjusting for the mothers' education level, the children's sex, birth weight, and time spent at the computer. The group of women who had not smoked during their pregnancy and had no STS exposure was used as the reference group. Subsequently, we conducted a stratified analysis where we compared the reference group of children whose mothers were well educated and smoked during pregnancy with children born to mothers with lower education levels. The effect of STS on wheezing and overweight in children was estimated as unadjusted and adjusted odds ratios with 95% confidence intervals (CI). All statistical analyses were performed using SPSS version 18.0 (SPSS Inc. Released 2009. PASW Statistics for Windows, Version 18.0. Chicago: SPSS Inc.).

## 3. Results

The women who participated in the study were highly educated; 73.1% of them had more than 10 years of education and a university degree. As many as 92.4% of the mothers reported never having smoked; however, 35.8% of them had been exposed to STS at home. Maternal smoking during pregnancy was associated with a younger age, lower education level, low socioeconomic status, a lower prevalence of breastfeeding, and postnatal antibiotic usage during the first year. Wheezing during the last 12 months in children was associated with a lower maternal education level, parental asthma, male sex, the number of siblings, and antibiotic use during the first year of life (Table 1). The prevalence of wheezing among children of nonsmoking mothers was 10.9% and among children born to mothers who were smoking during pregnancy was 15.9%. Among lower educated mothers it was 16.9% while in medium or high educated mothers it was 9.8%, crude OR 1.87, 95% CI 1.29–2.71.

The analysis of the distribution of risk factors for overweight showed that lower maternal education level, smoking during pregnancy, male sex, birth weight over 3500 g, and more than one hour per day spent at the computer increased the prevalence of overweight among 4–6-year-old children (Table 2). The prevalence of overweight among children of lower educated mothers was 11.7% while among medium or high educated mothers it was 6.3%, crude OR 2.00, 95% CI 1.30−3.00.

Table 3 shows the results of multivariate logistic regression models analyzing the association between maternal educations, STS, and wheezing among 4–6-year-old children. The multivariate model showed that, with reference to the group of nonsmoking women with a high education level and no STS exposure, STS exposure was found to be a statistically significant risk factor for wheezing in children born to mothers with lower education levels, after adjustment for antibiotic use during the first year of life, low birth weight, and child parity. Among nonsmokers mothers with lower education levels, the presence of household smoking was found to increase the risk of children wheezing to 1.96, 95% CI 1.28–2.98 and among smoking mothers to 2.12, 95% CI 1.04–4.35. The interaction term between lower education level, smoking, and children sex is not statistically significant (OR = 1.89, *P* = 0.20).

Table 4 presents the results of multivariate logistic regression models analyzing the association between maternal education levels, STS, and overweight in 4–6-year-old children. With reference to the group of high educated nonsmoking women with no STS exposure, the presence of STS exposure was found to be a significant risk factor for overweight in children, after adjusting for birth weight and time spent at the computer. The mothers' lower education levels and smoking during pregnancy, as well as STS exposure, increased the odds ratios of overweight in children to 3.57, 95% CI 1.76–7.21.

## 4. Discussion

This study demonstrated that STS increased the risk of wheeze for 4–6-year-old children who were not exposed (the mother was a nonsmoker) or exposed (the mother was a smoker) to maternal tobacco smoke while in utero; however, a statistically significant effect was evident only for children born to mothers with lower education levels (OR 1.96 and 2.12, accordingly). Using well-educated nonsmoking mothers not exposed to STS as a reference group, we did not find that household STS exposure had any statistically significant effect on the risk of wheezing in children of mothers with higher education levels. The effect of tobacco smoke on overweight was also higher among children of mothers with lower education levels. The odds ratio for overweight associated with tobacco smoke exposure was 3.57.

In this study, maternal smoking status was dichotomized to smoking and nonsmoking. The mothers who smoked during pregnancy tended to be different from nonsmokers in variables that also predicted the risk of child wheezing and overweight. In general, smokers were younger, were less educated, had a lower socioeconomic status, and were less likely to breastfeed, and their children more often used antibiotic during the first year of life. We found that among 4–6-year-old children of these women, maternal smoking status and STS had a relation with both wheezing and overweight, which was independent of maternal educational level, maternal age, and other variables that we adjusted for.

For the interpretation of this study, a few issues should be taken into account. Using individual data, we addressed possible confounding variables in multivariate analyses and estimated the effects of STS exposure on wheezing and overweight separately for the groups of children born to nonsmoking and smoking mothers with higher and lower education levels. Data on wheezing and overweight were obtained from parental reports through a questionnaire; the evaluation of exposure to tobacco smoke was indirect, and thus the possibility of random reporting bias exists. However, in this study, we controlled for the main variables that might confound the association between tobacco smoke exposure and children's wheezing and overweight—education level and first-year postnatal antibiotic use among them which could attenuate the strengths of the observed associations. Because of the low prevalence of maternal smoking during pregnancy, the sample size was insufficient to obtain statistically significant results during stratified analyses for the some estimation. Our similar estimates for unadjusted and adjusted associations suggest that sociodemographic and other differences between smokers and nonsmokers did not explain the observed association. These data are consistent with those obtained in studies from other countries [8, 31, 39, 40].

The results of our study are in accordance with those from other studies regarding the observed effects of tobacco smoke on the risk of overweight. As in other studies, lower maternal educational level [41, 42] and longer duration of children spent watching television and playing electronic games [43, 44] were associated with risk of being overweight. The observed association between smoking early in pregnancy and childhood obesity could be explained through metabolism disorder produced by tobacco smoke, which can result in reduced blood supply to the fetus because of the constrictive effects of blood vessels on maternal and uteroplacental blood supply [8].

Selection bias within the study population with such a difference in respondents and in nonrespondents groups should not lead to systematic bias because of the absence of a systematic difference in birth outcomes between the two response groups. Although we did adjust our analysis for proxy indicators of wheezing and overweight in children, we cannot exclude that residual noncontrolled confounding variables may affect the observed higher risk for wheezing and overweight in exposed children of mothers with lower education levels because of less healthy lifestyle habits [26]. This study did not include measures linked to genetic factors that are strongly associated with cigarette smoking. Therefore, there may well be residual genetic confounding that links the response to early-life tobacco smoke exposure with allergies and metabolic disorders later in life. The results of our previously published study suggested that smoking, even at low levels, ought to be considered a potential risk factor for adverse birth outcomes and that genetic polymorphism may contribute to individual variation in response to tobacco smoke [45].

A variety of adverse health outcomes are linked to cigarette smoking before and during pregnancy, and there are no speculations about the possible difference in health effects by children's sex. Maternal prenatal cigarette smoke damages the antioxidant system, has a negative impact on both the mother and the fetus on the genetic and cellular levels, and might disturb the development of the immune system in the fetus [1, 46]. Genetic polymorphism associated with airway hyperresponsiveness also has an impact on the expression of asthma symptoms [18, 47].

There is evidence that the impact of tobacco smoke on wheezing and being overweight in individual children is not on a par and that individual genetic predisposition may have an impact on the prognosis of children's health [16, 17, 26, 27]. Previous studies have suggested several plausible explanations for the gene-smoking interaction. Glutathione S-transferase (GST) M1 enzyme product that is involved in detoxification of both reactive tobacco metabolic intermediates and reactive oxygen species (GSTM1) null genotype has been shown to modify the effects of fetal tobacco smoke exposure on childhood asthma and wheezing [20, 48–50]. It has also been shown that maternal smoking during pregnancy is related to changes in DNA methylation [51, 52]. However, whether these changes underlie the associations between tobacco smoke exposure and children's obesity remains vague [32].

In conclusion, the mechanisms by which maternal smoking during pregnancy and STS may program children's postnatal health need to be studied further. Our results suggested that exposure to active maternal smoking during fetal development as well as later STS exposure led to an increased risk for wheezing and obesity in 4−6-year-old children. These results underlined the importance of health care interventions helping to quit smoking prior to conception and that targeted interventions must comprise cessation of household smoking for the prevention of chronicle diseases such as allergies and obesity in their children. Future studies are needed in children to identify the associations and interactions of socioenvironmental factors, tobacco smoke, and genetic predisposition to allergy and metabolic disorders.

## Figures and Tables

**Table 1:** Distribution of variables according to wheezing during the last 12 months among children and unadjusted effects as odds ratios (OR) and 95% confidence intervals (CI).

Variables	Wheezing yes *N* (%)	Wheezing no *N* (%)	Odds ratios∗∗ 95% CI
Mother's age at childbirth (years)			
<30	110 (11.1%)	885 (88.9%)	1
31 and more	58 (11.7%)	436 (88.3%)	1.07 (0.75–1.52)
Maternal education level			
Low (10 or less years)∗	52 (16.9%)	255 (83.1%)	1.87 (1.29–2.71)
Medium, high (>10 years)	116 (9.8%)	1066 (90.2%)	1
Socioeconomic status			
Low	50 (12.5%)	351 (87.5%)	1.17 (0.81–1.69)
Moderate, high	118 (10.8%)	970 (89.2%)	1
Maternal smoking during pregnancy			
No	150 (10.9%)	1226 (89.1%)	1
Yes	18 (15.9%)	95 (84.1%)	1.55 (0.88–2.71)
Maternal secondhand smoking			
No	98 (10.3%)	858 (89.7%)	1
Yes	70 (13.1%)	463 (86.9%)	1.32 (0.94–1.86)
Gas cooking			
No	52 (9.7%)	483 (90.3%)	1
Yes	116 (12.2%)	838 (87.8%)	1.29 (0.90–1.84)
Children's sex			
Male∗	103 (14.0%)	635 (86.0%)	1.71 (1.22–2.41)
Female	65 (8.7%)	686 (91.3%)	1
Children parity			
1	79 (9.6%)	743 (90.4%)	1
2 and more∗	89 (13.3%)	578 (86.7%)	1.45 (1.04–2.02)
Birth weight			
<2500 g	14 (15.4%)	77 (84.6%)	1.47 (0.77–2.74)
2501 g and more	154 (11.0%)	1244 (89.0%)	1
Breastfeeding			
No	16 (16.2%)	83 (83.8%)	1.57 (0.86–2.83)
Yes	152 (10.9%)	1238 (89.1%)	1
Allergy			
No	61 (5.9%)	973 (94.1%)	1
Yes∗	107 (23.5%)	348 (76.5%)	4.90 (3.45–6.97)
Eczema			
No	148 (10.7%)	1240 (89.3%)	1
Yes∗	20 (19.8%)	81 (80.2%)	2.07 (1.19–3.57)
Paracetamol use during the first year of life			
No	41 (9.1%)	412 (90.9%)	1
Yes	127 (12.3%)	909 (87.7%)	1.40 (0.95–2.07)
Antibiotic use during the first year of life			
No	75 (7.7%)	899 (92.3%)	1
Yes∗	93 (18.1%)	422 (81.9%)	2.64 (1.88–3.71)
Asthma in parents			
No	140 (10.1%)	1251 (89.9%)	1
Yes∗	28 (28.6%)	70 (71.4%)	3.57 (2.17–5.87)

*P < 0.05.

∗∗Unadjusted associations are presented as wheezing odds ratio (OR) with 95% confidence intervals of univariate analysis.

**Table 2:** Distribution of variables according to children's overweight status and unadjusted effects as odds ratios (OR) and 95% confidence intervals (CI).

Risk factors	Overweight yes *N* (%)	Overweight no *N* (%)	Odds ratios∗∗ 95% CI
Mothers' age at childbirth (years)			
<30	79 (7.9%)	916 (92.1%)	1.25 (0.81–1.91)
31 and more	32 (6.5%)	426 (93.5%)	1
Maternal education level			
Low (10 or less years)∗	36 (11.7%)	271 (88.3%)	2.00 (1.30–3.00)
Medium, high (>10 years)	75 (6.3%)	1107 (93.7%)	1
Maternal active smoking∗			
No	88 (6.8%)	1212 (93.2%)	1
Yes	23 (12.2%)	166 (87.8%)	1.91 (1.17–3.11)
Maternal smoking during pregnancy∗			
No	94 (6.8%)	1282 (93.2%)	1
Yes	17 (15.0%)	96 (85.0%)	2.42 (1.38–4.21)
Maternal second-hand smoking			
No	68 (7.1%)	888 (92.9%)	1
Yes	43 (8.1%)	490 (91.9%)	1.15 (0.77–1.71)
Children's sex			
Male∗	65 (8.8%)	673 (91.2%)	1.48 (1.00–2.19)
Female	46 (6.1%)	705 (93.9%)	1
Breastfeeding			
No	10 (10.1%)	89 (89.9%)	1.43 (0.72–2.84)
Yes	101 (7.3%)	1289 (92.7%)	1
Paracetamol use during the first year of life			
No	26 (5.7%)	427 (94.3%)	1
Yes	85 (8.2%)	951 (91.8%)	1.47 (0.93–2.31)
Antibiotic use during the first year of life			
No	66 (6.8%)	908 (93.2%)	1
Yes	45 (8.7%)	470 (91.3%)	1.32 (0.89–1.96)
Birth weight∗			
<2500 g	6 (6.8)	82 (93.2%)	1.17 (0.48–2.84)
2500–3500 g	43 (5.9%)	688 (94.1%)	1
>3500 g	62 (9.3%)	608 (90.7%)	1.63 (1.09–2.44)
Watching TV			
≤1 h/day	36 (6.1%)	558 (93.9%)	1
>1 h/day	75 (8.4%)	820 (91.6%)	1.42 (0.94–2.14)
Time spent at the computer∗			
≤1 h/day	72 (6.3%)	1066 (93.7%)	1
>1 h/day	39 (11.1%)	312 (88.9%)	1.58 (2.84–6.47)

*P < 0.05.

∗∗Unadjusted associations are presented as overweight odds ratio (OR) with 95% confidence intervals of univariate analysis.

**Table 3:** Association of maternal education level, second-hand tobacco smoke (STS), and wheezing in 4–6-year-old children with reference to well-educated nonexposed to tobacco smoke mothers.

Maternal education level and smoking	Wheezing cases *N* (%)	Adjusted risk of wheezing∗ aOR (95% CI)
Mother nonsmoker		
High and no STS∗∗	72 (8.9%)	1 (reference)
High and STS	37 (11.6%)	1.32 (0.86–2.02)
Low and STS	41 (16.5%)	1.96 (1.28–2.98)
Mother smoker		
High and no STS∗∗	72 (8.9%)	1 (reference)
High and STS	7 (12.7%)	1.26 (0.54–2.93)
Low and STS	11 (19.0%)	2.12 (1.04–4.35)

*Results of multivariate logistic regression models are presented as associations of wheezing odds ratio (OR) with 95% confidence intervals adjusting for first-year postnatal antibiotic use, low birth weight, and child parity. ∗∗Reference category is high educated, nonsmokers, and nonexposed to second-hand tobacco smoke (STS) mothers. SES-specific STS effect on children wheezing is presented in nonsmoker mothers and smoker mothers by educational level.

**Table 4:** Association of maternal education level, second-hand tobacco smoke (STS), and overweight in 4–6-year-old children with reference to well-educated nonexposed to tobacco smoke mothers.

Maternal education level and smoking	Overweight cases *N* (%)	Adjusted risk of overweight∗ aOR (95% CI)
Mother nonsmoker		
High and no STS∗∗	53 (6.6%)	1 (reference)
High and STS	17 (5.3%)	0.80 (0.46–1.41)
Low and STS	24 (9.6%)	1.40 (0.84–2.34)
Mother smoker		
High and no STS∗∗	53 (6.6%)	1 (reference)
High and STS	5 (9.1%)	1.29 (0.49–3.41)
Low and STS	12 (20.7%)	3.57 (1.76–7.21)

*Results of stratified multivariate logistic regression models are presented as associations of overweight odds ratio (OR) with 95% confidence intervals adjusting for first-year postnatal antibiotic use, low birth weight, and time spent at the computer. ∗∗Reference category is high educated, nonsmokers, and nonexposed to second-hand tobacco smoke (STS) mothers. SES-specific STS effect on overweight is presented in nonsmoker mothers and smoker mothers by educational level.
